# Texas and the New Federal Quarantine Law

**Published:** 1906-08

**Authors:** 


### Texas and the New Federal Quarantine Law.

AUSTIN, TEXAS, July 18, 1906.

*Dr. Geo. R. Tabor, State Health Officer, Austin, Texas.*

DEAR DOCTOR: Will you please give me information on the following points for publication in the next issue of the TEXAS

MEDICAL JOURNAL, and greatly oblige. Please answer fully, and return this letter with your reply: When does the new Federal Quarantine Law, approved June 29th, go into effect? How and to what extent does it affect Texas; what changes will it make in the matter of State quarantine? Will Texas turn over to the Marine Hospital and Public Health Service the gulf quarantine stations, and discontinue inspections of vessels at those stations? Will Texas discontinue the inspection stations on the Rio Grande? State full extent to which Texas will be relieved of quarantine duty, and what amount of money will be thereby saved to the State. Any other information in this connection will be thankfully received.

Thanking you in advance, I am, sir,

Very truly yours,

F. E. DANIEL, M. D.,

Editor and Publisher TEXAS MEDICAL JOURNAL.

DEPARTMENT OF PUBLIC HEALTH AND VITAL STATISTICS.
STATE OF TEXAS.

AUSTIN, TEXAS, July 30, 1906.

*Dr. F. E. Daniel, Editor Texas Medical Journal, Austin, Texas.*

DEAR DOCTOR: Replying to your letter of the 18th, in which you ask questions with reference to the new Federal Quarantine Law, I beg to say that I can not give you any satisfactory answer for the reason that neither the Governor nor myself has the authority to turn over the Texas quarantine stations on the gulf coast and Rio Grande border to the Federal government. This matter will require an act of the Legislature, which meets in January.

Under this law the State can still maintain its quarantine stations along with the Federal stations, but as this would give a double service, working a hardship on the shipping and traveling public where there may be two sets of regulations to pass, the Legislature may, for this reason, see fit to relinquish State quarantine to the United States. Of course, if the State quarantine is turned over to the Federal government, it will not then be necessary to make appropriations for salaries for the State quarantine officers, which will mean a saving of several thousand dollars in salaries to the State.

As the new Federal quarantine law provides that the Secretary of the Treasury may purchase all the stations and equipment, I judge there will be no interference with the present system until the next Legislature meets, and either authorizes the sale of the stations to the Federal government, or declines to authorize this sale and continue the State quarantine independent of the Federal service.

I will be glad to give you any other information in connection with this that I may have.

Very truly yours,
GEORGE R. TABOR,
State Health Officer.

